# Comparative Evaluation of Augmentation Stability of L-Shaped Collagenated Soft Block Bone with Physically Crosslinked and Non-Crosslinked Collagen Membranes: A Retrospective Observational Cohort Study

**DOI:** 10.3390/diagnostics16111675

**Published:** 2026-05-29

**Authors:** Jae-Hong Lee, Hyeok-Jun Yang, Nguyen Thi Phuong Thao

**Affiliations:** 1 Department of Periodontology, College of Dentistry and Institute of Oral Bioscience, Jeonbuk National University, Jeonju 54896, Republic of Korea; gurwns7224@naver.com (H.-J.Y.); phuongthaorhm@gmail.com (N.T.P.T.); 2 Research Institute of Clinical Medicine of Jeonbuk National University-Biomedical, Research Institute of Jeonbuk National University Hospital, Jeonju 54907, Republic of Korea; 3 Faculty of Odonto-Stomatology, Hue University of Medicine and Pharmacy, Hue University, Hue City 530000, Vietnam

**Keywords:** bone regeneration, bone substitute, collagen, dental implants, xenografts

## Abstract

**Background/Objectives**: The aim of this study was to compare the augmentation stability and clinical outcomes of L-shaped collagenated soft block bone substitutes (BBS) used in combination with either a self-assembly technology (SAT)-based physically crosslinked resorbable collagen membrane (RCM) or a conventional non-crosslinked RCM for peri-implant dehiscence defects. **Methods**: This retrospective cohort study included 30 patients who underwent guided bone regeneration (GBR) with simultaneous implant placement. The patients were treated with either a physically crosslinked membrane (PCM group, *n* = 15) or a non-crosslinked membrane (NCM group, *n* = 15). Clinical, radiographic, and profilometric parameters were evaluated at baseline, immediately post-GBR, and at re-entry surgery. Early wound healing complications and patient-reported outcomes were also assessed. **Results**: Both groups achieved significant defect resolution without severe adverse events. The mean reductions in defect width and height were 4.47 ± 1.82 mm (92.9%) and 4.07 ± 2.19 mm (89.4%) in the PCM group and 3.80 ± 1.59 mm (89.5%) and 4.13 ± 1.64 mm (86.9%) in the NCM group, respectively. Both groups showed comparable dimensional changes in hard and soft tissues, with no statistically significant differences in radiographic or profilometric outcomes. The incidence of wound healing complications, as well as patient-reported postoperative pain and swelling, were similar between the groups. **Conclusions**: Within the limitations of this retrospective pilot cohort study, SAT-based physically crosslinked RCMs used in combination with L-shaped soft BBS demonstrated clinical, radiographic, profilometric, and patient-reported outcomes similar to those observed with conventional non-crosslinked RCMs, without major short-term postoperative complications. These preliminary findings suggest that SAT-based RCMs may represent a feasible membrane option for GBR; however, these findings should be interpreted as preliminary and hypothesis-generating and should be confirmed in larger, adequately powered prospective clinical studies.

## 1. Introduction

Dental implants are widely accepted as the standard treatment for partial or complete edentulism [1]. However, guided bone regeneration (GBR) is frequently required to correct osseous defects and achieve optimal functional and esthetic prosthetic outcomes [2]. Although various surgical techniques and biomaterials have been proposed to enhance the predictability of GBR, successful regeneration relies on the following key principles: primary tension-free wound closure, adequate angiogenesis, space maintenance, and the stability of the wound and the implant [3]. Nevertheless, a universally accepted “gold standard” protocol for ideal bone regeneration remains elusive, and the relative superiority of the diverse biomaterials and techniques currently employed in clinical practice continues to be a subject of debate [4,5].

Particulate bone substitutes (PBS), commonly used in conventional GBR, are susceptible to collapse under soft tissue pressure during the healing phase, often resulting in inadequate maintenance of the graft volume [6,7]. Although autogenous bone blocks offer superior volumetric stability compared to PBS, their use is limited by donor site morbidity and potential complications such as wound infection, sensory disturbances, and hemorrhage [8,9]. To overcome these limitations, collagenated soft block bone substitutes (BBS) were introduced, offering improved physical and mechanical handling properties. Notably, in vitro and clinical investigations indicated that L-shaped, properly contoured soft BBS confer distinct clinical advantages, particularly by facilitating surgical handling and ensuring predictable stability in horizontal and vertical augmentation [10,11,12].

Resorbable collagen membranes (RCMs) are widely employed in GBR. Physically crosslinked RCMs, recognized for their superior biocompatibility and reduced complication rates, are increasingly favored over chemically crosslinked RCMs, which have been associated with a higher incidence of adverse tissue reactions and compromised bone regeneration [13,14]. Despite these advancements, there is limited evidence regarding the augmentation stability of L-shaped soft BBS when combined with physically crosslinked RCMs compared to non-crosslinked RCMs. We hypothesized that self-assembly technology (SAT)-based physically crosslinked RCM can provide superior dimensional stability compared to a conventional non-crosslinked RCM or, at minimum, demonstrate clinical outcomes with an improved safety profile comparable to chemically crosslinked alternatives. Therefore, this retrospective study aimed to investigate whether the application of L-shaped soft BBS in combination with physically crosslinked RCMs offers advantages in clinical, radiographic, profilometric, and patient-reported outcomes relative to their use with non-crosslinked RCMs.

## 2. Materials and Methods

### 2.1. Ethics and Study Design

The study protocol was established in accordance with the revised Declaration of Helsinki and adhered to the Strengthening the Reporting of Observational Studies in Epidemiology (STROBE) guidelines [15,16]. This retrospective study was approved by the Institutional Review Board of Jeonbuk National University Hospital (Approval No. CUH 2025-09-014), and the requirement for written informed consent was waived owing to the retrospective nature of the study and the use of anonymized clinical data.

### 2.2. Patient Selection

This retrospective cohort study analyzed clinical, radiographic, and profilometric data obtained from patients who underwent implant surgery at the Department of Periodontology, Jeonbuk National University Dental Hospital between May 2024 and July 2025. The inclusion criteria were as follows: (1) presence of buccal dehiscence defects (horizontal and vertical dimensions > 1 mm) requiring bone augmentation following implant placement; (2) implant surgery involving simultaneous GBR using L-shaped soft BBS in combination with an RCM; and (3) adequate oral hygiene (Full Mouth Bleeding Score and Full Mouth Plaque Score < 25%) and systemic health (American Society of Anesthesiologists [ASA] physical status class I or II). Patients were excluded if they were heavy smokers (≥20 cigarettes/day) or presented with uncontrolled oral or systemic diseases. Because of the retrospective observational design, patients were not prospectively randomized or assigned to either treatment group. Group classification was based on the type of RCM actually used during routine clinical care. After application of the predefined inclusion and exclusion criteria, a total of 30 patients were included in the final analysis, comprising 15 patients in the PCM group and 15 patients in the NCM group.

### 2.3. Study Size Rationale

As this was a retrospective exploratory pilot cohort study, no formal hypothesis-based sample size calculation was performed. The study size was determined by feasibility and the availability of all eligible consecutive cases treated within the predefined study period from May 2024 to July 2025. Because prior clinical data were unavailable for the specific combination of a SAT-based physically crosslinked RCM and L-shaped soft BBS, this study was intended to provide preliminary estimates of clinical outcomes, dimensional changes, variability, and early complications. Therefore, the sample size was considered appropriate for an exploratory pilot study but not powered to establish superiority, equivalence, or formal non-inferiority between the two membrane types.

### 2.4. Surgical Procedures

**Figure 1:**
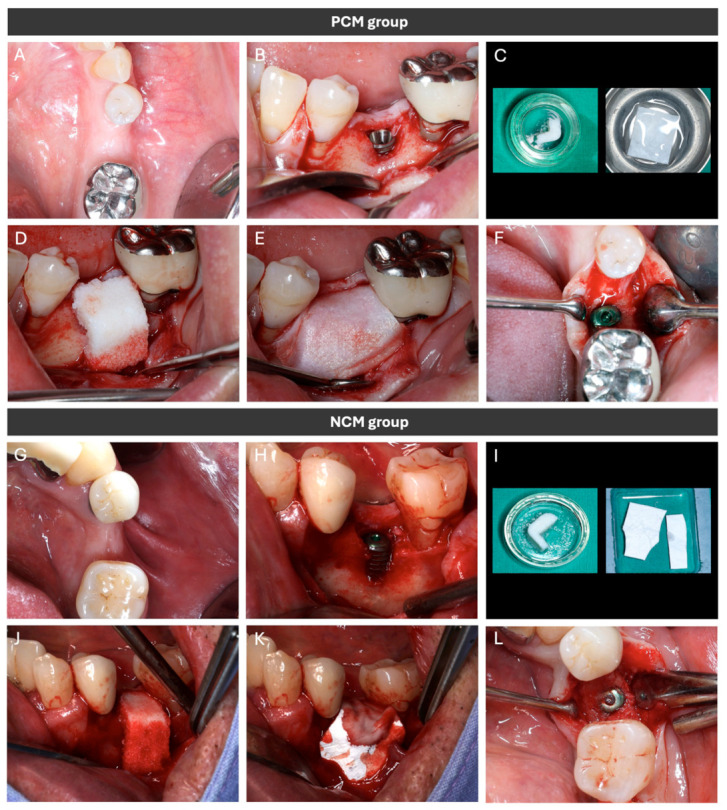
Representative clinical photographs illustrating the surgical sequence. (**A**,**G**) Preoperative facial views. (**B**,**H**) Buccal dehiscence defects visible following implant placement. (**C**,**I**) Preparation of the L-shaped trimmed collagenated soft block bone substitutes (BBS) with a physically crosslinked resorbable collagen membrane (PCM) in (**C**) and a non-crosslinked resorbable collagen membrane (NCM) in (**I**). (**D**,**J**) Adaptation of the L-shaped soft BBS to the defects. (**E**,**K**) The augmented sites covered with either the PCM or NCM. (**F**,**L**) Occlusal views during re-entry surgery.

The surgical procedures were performed by a single board-certified periodontist (JHL). Following the elevation of a full-thickness mucoperiosteal flap, a bone-level tapered implant fixture with a sandblasted, large-grit, acid-etched surface (KS^®^ SA, Osstem, Seoul, Republic of Korea) was placed in the optimal prosthetically driven position according to the manufacturer’s instructions. A deproteinized porcine bone mineral containing 10% collagen (THE Graft^®^ Collagen, Purgo Biologics, Seongnam, Republic of Korea) was trimmed into an “L”-shaped configuration using a #15 surgical blade to precisely adapt to the buccal dehiscence defect. The graft was contoured to extend at least 1 mm beyond the defect margins buccally and vertically (Figure 1). For analysis, the cohort was retrospectively classified according to the membrane type used during surgery, as follows:

- Physically crosslinked membrane group (PCM group): A non-chemically, physically crosslinked RCM fabricated via SAT (THE Cover^®^, Purgo Biologics) was utilized. The membrane was positioned to extend at least 1 mm beyond the augmented site to ensure stable adaptation and complete coverage of the defect.
- Non-crosslinked membrane group (NCM group): A conventional native non-crosslinked RCM (Bio-Gide^®^, Geistlich Pharma AG, Wolhusen, Switzerland) was applied over the augmented site following the identical protocol used in the PCM group.

When necessary, a periosteal vertical mattress suture was utilized to stabilize the membrane. Tension-free primary closure was achieved using a combination of interrupted and horizontal mattress sutures with 3-0 and 4-0 e-PTFE (Biotex^®^, Purgo Biologics) and 5-0/6-0 nylon sutures (Ethilon^®^, Ethicon, Johnson & Johnson Medical GmbH, Norderstedt, Germany), which are non-resorbable.

### 2.5. Postsurgical Care

Patients were instructed to maintain proper oral hygiene at home. The postoperative medication regimen followed the institutional protocol used for implant surgery with simultaneous GBR and was based on previously reported postoperative protocols in implant dentistry. Amoxicillin (500 mg) and ibuprofen (200 mg) were administered three times daily for five to seven days, unless contraindicated [17,18]. In addition, a 0.12% chlorhexidine mouthrinse was prescribed twice daily for two weeks to support postoperative plaque control and reduce the risk of wound contamination. Sutures were removed two weeks after surgery, and re-entry surgery was performed five months after implant placement.

### 2.6. Outcome Parameters

Clinical outcomes were assessed by the operating surgeon (JHL), while radiographic, profilometric, and patient-reported outcomes were evaluated by a single, blinded, and calibrated examiner not involved in the surgery. Measurements were recorded at three time points: baseline (T0), immediately after GBR (T1), and at re-entry surgery (T2). Intra-examiner calibration was performed, yielding an intra-class correlation coefficient > 0.90, indicating high reliability.

#### 2.6.1. Clinical Measurements

Defect width (DW) was defined as the linear distance between the widest mesial and distal points of the dehiscence, and defect height (DH), the distance from the implant platform to the first bone-to-implant contact. Both parameters were recorded to the nearest millimeter at T1 and T2 using a manual periodontal probe (CP-15 UNC, Hu-Friedy, Chicago, IL, USA).

#### 2.6.2. Radiographic Measurements

Sagittal cone-beam computed tomography scans were analyzed using 3D dental imaging software (OnDemand 3D^®^, ver. 1.0.10.7510, Cybermed, Seoul, Republic of Korea). Horizontal reference lines perpendicular to the long axis of the implant were established. Hard tissue thickness was measured at the implant shoulder (HT0), at 1 mm (HT1) and 2 mm (HT2) apical to the shoulder, and at 45° (45-HT) and 90° (90-HT) angulations from the shoulder axis at T1 and T2.

#### 2.6.3. Profilometric Measurements

To assess soft tissue profile changes, digital impressions were obtained using an intraoral scanner (Medit i700 wireless^®^, Medit, Seoul, Republic of Korea), generating stereolithography (STL) files at T0, T1, and T2. Using 3D analysis software (Medit Link^®^, ver. 3.3.2, Medit; Geomagic Control X^®^, ver. 2023.3.0, 3D Systems, Rock Hill, SC, USA), horizontal reference lines were established on the sagittal plane of the STL models. Soft tissue thickness was measured at the implant shoulder (ST0), 1 mm (ST1), and 2 mm (ST2) apical to the shoulder, and at 45° (45-ST) and 90° (90-ST) angulations.

#### 2.6.4. Early Wound Healing Complications and Postoperative Discomfort

Early wound healing complications (membrane exposure, graft exposure, soft tissue dehiscence) were evaluated at two weeks post-surgery. Patient-reported outcome measures, including the severity and duration of pain and swelling, were assessed via a self-administered questionnaire. A visual analog scale ranging from 0 (no pain/swelling) to 10 (worst imaginable pain/swelling) was used for quantification.

### 2.7. Statistical Analysis

Descriptive statistics, including means, standard deviations, medians, and frequencies, were calculated. The normality of continuous variables and homogeneity of variance were assessed using the Shapiro–Wilk and Levene’s tests, respectively. As these assumptions were satisfied, continuous variables were compared between the groups using independent *t*-tests. Categorical variables were analyzed using the chi-square test or Fisher’s exact test, as appropriate. Given the exploratory pilot design and the assessment of multiple clinical, radiographic, profilometric, wound-healing, and patient-reported outcomes, *p*-values were interpreted descriptively. The main clinical dimensional outcomes were considered the primary focus of interpretation, whereas the remaining outcomes were regarded as secondary exploratory outcomes. To reduce the risk of type I error, the Benjamini–Hochberg false discovery rate procedure was additionally applied to secondary intergroup comparisons as a sensitivity analysis. Statistical significance was set at *p* < 0.05. The analyses were performed using statistical software packages (SPSS^®^, version 28; IBM Corp., Armonk, NY, USA; JMP^®^, version 17.1; SAS Institute, Cary, NC, USA; MedCalc^®^, version 23.2.6; MedCalc Software Ltd., Mariakerke, Belgium).

## 3. Results

### 3.1. Baseline Characteristics

A total of 30 patients, comprising 13 men (43.3%) and 17 women (56.7%) with a mean age of 60.7 ± 13.5 years (range, 30–81 years), were included in the analysis. Of these, 15 patients (mean age: 62.7 ± 11.9 years) were assigned to the PCM group and 15 (mean age: 58.7 ± 15.5 years) were assigned to the NCM group. No patient experienced serious adverse events, such as severe or uncontrolled peri-implant infections, during the hard tissue healing period. Furthermore, there were no statistically significant differences in baseline demographic characteristics between both groups (Table 1).

### 3.2. Clinical Outcomes

In the PCM group, the mean reduction in DW from T1 to T2 was 4.47 ± 1.82 mm, corresponding to a defect resolution of 92.9%. Similarly, the mean reduction in DH was 4.07 ± 2.19 mm (89.4%). The NCM group showed comparable outcomes, with mean reductions of 3.80 ± 1.59 mm (89.5%) for DW and 4.13 ± 1.64 mm (86.9%) for DH. No statistically significant intergroup differences were observed for any of the clinical parameters (*p* > 0.05). Although no significant intergroup differences were observed, it is noteworthy that both treatment modalities achieved substantial defect resolution (>86% reduction in defect height and width), confirming the clinical efficacy of the L-shaped soft BBS technique regardless of the membrane type used (Table 2).

### 3.3. Radiographic and Profilometric Outcomes

**Figure 2:**
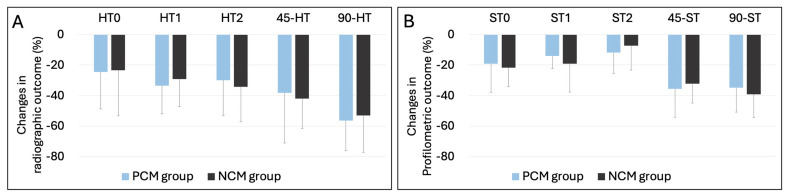
Comparative analysis of clinical, radiographic, and profilometric outcomes. Changes in (**A**) cone-beam computed tomography-based radiographic outcomes, and (**B**) intraoral scan-based profilometric outcomes were assessed from implant surgery with guided bone regeneration to re-entry surgery. No statistically significant differences were observed between both groups in any of the radiographic or profilometric parameters (*p* > 0.05 for all).

In the radiographic assessment, the PCM group exhibited a mean hard tissue thickness reduction of 1.10 ± 0.78 mm (36.5% reduction) from T1 to T2. The profilometric analysis revealed a mean soft tissue thickness reduction of 1.30 ± 1.07 mm (23.1%). In the NCM group, the corresponding reductions were 1.25 ± 1.00 mm (36.4%) for hard tissue and 1.41 ± 1.22 mm (23.9%) for soft tissue. These dimensional changes in hard and soft tissues were not statistically significantly different between the groups (*p* > 0.05) (Figure 2).

### 3.4. Complications and Discomfort Outcomes

**Figure 3:**
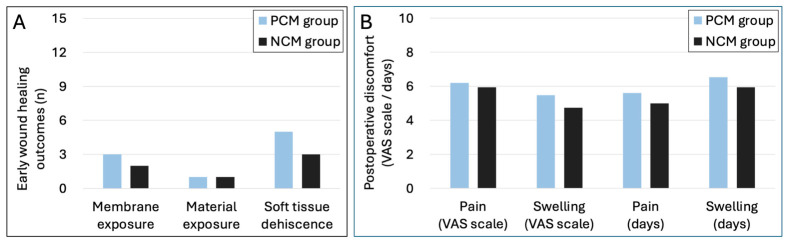
Assessment of early wound healing and patient-reported outcomes. (**A**) Incidence of early wound healing complications observed at two weeks post-surgery. (**B**) Patient-reported outcome including the severity and duration of postoperative pain and swelling. No statistically significant differences were detected between the PCM and NCM groups (*p* > 0.05).

No serious adverse events, such as severe or uncontrolled peri-implant infections, occurred in either group during the healing period. The incidence of early wound healing complications did not differ significantly between the groups (*p* > 0.05). Specifically, in the PCM group, membrane exposure, graft material exposure, and soft tissue dehiscence were observed in three (20.0%), one (6.7%), and five (33.3%) patients, respectively. In the NCM group, these complications occurred in two (13.3%), one (6.7%), and three (20.0%) patients, respectively. Regarding patient-reported outcome measures, there were no significant intergroup differences in the severity of postoperative pain (PCM: 6.2 ± 2.0; NCM: 5.9 ± 2.0) or swelling (PCM: 5.5 ± 1.9; NCM: 4.7 ± 1.8). Similarly, the duration of pain (PCM: 5.6 ± 3.4 days; NCM: 5.0 ± 2.1 days) and swelling (PCM: 6.5 ± 5.9 days; NCM: 5.9 ± 2.9 days) were comparable between the groups (all *p* > 0.05) (Figure 3).

## 4. Discussion

This retrospective cohort study evaluated the dimensional stability of L-shaped collagenated soft BBS used in combination with either a SAT-based physically cross-linked RCM or a conventional non-crosslinked RCM. Both treatment modalities demonstrated favorable clinical, radiographic, and profilometric outcomes, with no major postoperative complications, supporting the clinical feasibility of the L-shaped soft BBS technique and the potential applicability of SAT-based RCMs in GBR. However, because this was an exploratory pilot study, the absence of statistically significant inter-group differences should not be interpreted as definitive evidence of equivalence. The observed between-group differences in defect width and height reduction were small (0.67 mm and −0.07 mm, respectively); nevertheless, clinically relevant small-to-moderate differences cannot be fully excluded in this limited pilot dataset.

Although chemical crosslinking strategies like carbodiimide treatment or glutaraldehyde fixation establish strong covalent intermolecular bonds to enhance resistance to enzymatic degradation, physical crosslinking methods such as ultraviolet (UV) irradiation, dehydrothermal (DHT) treatment, and SAT offer distinct safety advantages [19,20]. Although chemical approaches provide durability, they have been associated with potential cytotoxicity, inflammatory responses, and impaired tissue integration due to residual reagents. In contrast, physical techniques obviate the need for chemical agents, thereby preserving the ultrastructure and intrinsic biocompatibility of collagen. This renders them particularly suitable for GBR and soft tissue engineering [19,20]. However, although UV and DHT treatments achieve moderate stabilization, their limited long-term dimensional stability underscores the need for more robust alternatives [21].

In this context, SAT has emerged as a promising approach that exploits the inherent ability of collagen triple helices to organize into dense fibrillar networks under controlled physicochemical conditions [22]. This self-assembly process enhances tensile strength and delays enzymatic breakdown while maintaining favorable biological compatibility [22,23]. Importantly, SAT-generated membranes recapitulate the ultrastructural architecture of the native extracellular matrix, thereby facilitating cell adhesion, vascular ingrowth, and subsequent bone regeneration [22,23]. Although SAT may provide comparatively lower resistance to degradation compared to chemical crosslinking, its superior safety profile and physiologic tissue response suggest a distinct clinical advantage in GBR procedures, where the optimal balance between membrane stability and biocompatibility is critical. Future well-designed clinical trials are warranted to confirm the specific efficacy of SAT-based membranes, particularly in the reconstruction of large and complex defects.

In the present cohort, pooled analyses of the PCM and NCM groups revealed a progressive reduction in both hard and soft tissue thickness. Hard tissue changes were recorded as reductions of 0.84 ± 1.02 mm (24.0%) at HT0, 1.00 ± 0.74 mm (32.2%) at HT2, 1.23 ± 0.76 mm (40.0%) at 45-HT, and 1.83 ± 1.00 mm (54.6%) at 90-HT. Correspondingly, soft tissue thickness exhibited reductions of 1.05 ± 0.78 mm (20.5%) at ST0, 0.63 ± 0.71 mm (9.6%) at ST2, 1.72 ± 0.91 mm (33.9%) at 45-ST, and 2.34 ± 1.37 mm (37.0%) at 90-ST. These results corroborate previous evidence suggesting that L-shaped soft BBS provide superior dimensional stability compared with particulate grafts, particularly in peri-implant dehiscence defects [24,25]. Furthermore, these findings align with prior clinical studies demonstrating that L-shaped soft BBS achieve predictable horizontal and vertical augmentation, confirming favorable volumetric maintenance without significant adverse events [26]. Consequently, the inherent volumetric stability provided by the contoured L-shaped soft BBS likely played a dominant role in maintaining the graft space. This strong structural support from the bone substitute itself may have masked subtle differences in the barrier function between both membrane types, resulting in comparable clinical outcomes.

Comparison with a previous clinical study reveals that our findings indicate a greater magnitude of dimensional reduction at 45-HT and 90-HT. The earlier investigation reported reductions of 0.79 ± 0.54 mm (23.0%) at 45-HT and 0.77 ± 0.60 mm (25.5%) at 90-HT, considerably lower than those observed in the present study [11]. This discrepancy is most plausibly explained by differences in implant site distribution. Whereas the prior study was restricted to anterior regions, our cohort included anterior and posterior sites. Posterior sites are subject to greater occlusal loading and often present with less favorable soft tissue characteristics, making them more prone to pronounced dimensional alterations [27,28]. These observations suggest that although L-shaped soft BBS provide reliable volumetric stability, the extent of dimensional change is site-dependent, underscoring the clinical importance of implant location in anticipating treatment outcomes.

The relatively frequent occurrence of early wound healing complications in both groups deserves careful interpretation. In the present study, membrane exposure, graft exposure, and soft tissue dehiscence were observed in 20.0%, 6.7%, and 33.3% of patients in the PCM group and in 13.3%, 6.7%, and 20.0% of patients in the NCM group, respectively. Although no statistically significant intergroup differences were detected, these findings should not be interpreted as evidence of equivalent complication profiles because this exploratory pilot study was underpowered for categorical safety outcomes. From a clinical perspective, early membrane exposure and soft tissue dehiscence are relevant because they may increase bacterial contamination, compromise wound stability, reduce the effective barrier function of the membrane, and potentially impair the predictability of bone augmentation. Previous evidence suggests that membrane exposure after GBR can negatively affect augmentation outcomes, particularly in peri-implant dehiscence defects [9,29]. Nevertheless, in the present cohort, these complications were not accompanied by severe infection, uncontrolled inflammation, graft loss, or implant failure during the observed healing period.

Regarding postoperative discomfort and early wound healing complications, no severe adverse events were observed, and no clinically significant differences were detected between the PCM and NCM groups during the initial healing phase. Consistent with recent systematic reviews reporting that RCMs generally exhibit favorable safety profiles in GBR, these findings suggest that SAT-based physically cross-linked RCMs may have a short-term safety profile comparable to that of non-crosslinked RCMs. However, because this pilot study was not designed or powered as a formal non-inferiority trial, these findings should be interpreted cautiously as exploratory and hypothesis-generating rather than confirmatory [30,31]. Collectively, these results suggest that PCM provides comparable short-term clinical safety, while its biomimetic collagen architecture may offer theoretical long-term benefits by maintaining barrier function and structural stability without the use of cytotoxic reagents [21].

This study has some limitations. First, the retrospective and observational design is inherently subject to selection bias and uncontrolled confounding. Although baseline characteristics were comparable between groups, unmeasured factors may have influenced the outcomes. Second, the sample size was limited because this retrospective pilot cohort included all eligible consecutive cases treated within a predefined period. Although the inclusion of 15 patients per group was considered acceptable for a feasibility-oriented pilot investigation and for generating preliminary estimates of variance and complication rates, the study was underpowered to detect small-to-moderate intergroup differences. Accordingly, nonsignificant findings should not be interpreted cautiously and not as definitive evidence of equivalence or non-inferiority. Third, the observation period was restricted to the interval between GBR and re-entry surgery. Long-term follow-up assessing peri-implant bone stability, soft tissue contour, and esthetic outcomes is essential to establish the durability and long-term efficacy of SAT-based physically crosslinked RCMs. Another limitation is the non-random allocation of membrane type. Because patients were retrospectively classified according to the membrane used during routine clinical care, the possibility of selection bias or unmeasured confounding cannot be excluded. Although all surgeries were performed by a single experienced periodontist using a standardized surgical protocol and baseline characteristics were comparable between the groups, membrane selection based on clinical availability and routine material choice may have influenced the results, introducing the possibility of selection bias. Accordingly, the present findings should be interpreted cautiously as preliminary, feasibility-oriented observations. The nonsignificant intergroup differences should not be construed as evidence of equivalence, and the observed similarity between groups should be confirmed in larger, adequately powered prospective studies with longer follow-up.

## 5. Conclusions

Within the limitations of this retrospective exploratory pilot study, SAT-based physically crosslinked RCMs used in combination with L-shaped soft BBS were not associated with any apparent major short-term safety concerns and yielded clinical, radiographic, profilometric, and patient-centered outcomes similar to those observed with conventional non-crosslinked RCMs. These findings should be regarded as preliminary and hypothesis-generating rather than as definitive evidence of equivalence or broad clinical applicability. Well-designed, adequately powered prospective randomized controlled trials with longer-term follow-up are required to confirm the clinical effectiveness, dimensional stability, safety, and generalizability of SAT-based physically crosslinked RCMs in GBR procedures.

## Figures and Tables

**Table 1:** Demographic and baseline characteristics of the study population.

	PCM Group (*n* = 15)		NCM Group (*n* = 15)		
	*n*	%	*n*	%	*p*-Value
Sex					
Men	6	40.0	7	46.7	0.717
Women	9	60.0	8	53.3	
Age (years)					
Mean	62.7		58.7		0.441
Standard deviation	11.9		15.5		
Median	67.0		60.0		
Min.–Max.	44–81		30–81		
Diabetes mellitus					
Yes	2	13.3	2	13.3	1.000
Smoking status					
Non-smoker	14	93.3	13	86.7	0.549
Current smoker (<20 cigarettes/day)	1	6.7	2	13.3	
Location					
Maxillary anterior region	3	20.0	1	6.7	0.410
Maxillary premolar region	2	13.3	0	0.0	
Maxillary molar region	1	6.7	2	13.3	
Mandibular anterior region	1	6.7	1	6.7	
Mandibular premolar region	5	33.3	4	26.7	
Mandibular molar region	3	20.0	7	46.7	

NCM, non-crosslinked resorbable collagen membrane; PCM, non-chemically and inherent physically crosslinked resorbable collagen membrane.

**Table 2:** Clinical, radiographic, and profilometric outcomes assessed at implant surgery (T1) and re-entry surgery (T2).

		PCM Group		NCM Group		PCM vs. NCM Group	
Parameters (mm)	T1	T2	*p*-Value	T1	T2	*p*-Value	*p*-Value
Clinical outcomes							
DW	4.87 ± 1.88 [4.50 (4.00, 6.00)]	0.40 ± 0.81 [0.00 (0.00, 0.50)]	<0.001 **	4.23 ± 1.52 [4.00 (3.00, 5.00)]	0.43 ± 0.59 [0.00 (0.00, 0.75)]	<0.001 **	0.294
DH	4.53 ± 1.97 [4.00 (3.00, 5.25)]	0.47 ± 1.03 [0.00 (0.00, 0.50)]	<0.001 **	4.70 ± 1.39 [5.00 (4.00, 5.00)]	0.57 ± 0.70 [0.50 (0.00, 1.00)]	<0.001 **	0.925
Radiographic outcomes							
HT0	3.52 ± 0.80 [3.40 (3.13, 3.68)]	2.65 ± 0.99 [2.78 (2.09, 3.30)]	0.073	3.12 ± 0.74 [3.25 (2.42, 3.51)]	2.31 ± 1.01 [2.27 (1.85, 3.17)]	0.090	0.897
HT1	3.35 ± 0.58 [3.12 (3.04, 3.69)]	2.21 ± 0.67 [2.13 (1.80, 2.47)]	0.003 *	3.07 ± 0.97 [3.22 (2.47, 3.64)]	2.21 ± 1.14 [1.82 (1.45, 2.58)]	0.131	0.377
HT2	2.80 ± 0.38 [2.67 (2.50, 3.05)]	1.95 ± 0.68 [2.03 (1.45, 2.32)]	0.008 *	3.25 ± 0.38 [3.22 (3.08, 3.26)]	2.11 ± 0.71 [2.29 (1.76, 2.48)]	<0.001 **	0.459
45-HT	2.83 ± 0.53 [2.84 (2.34, 3.27)]	1.78 ± 0.95 [2.18 (1.06, 2.47)]	0.016 *	3.36 ± 0.83 [3.28 (3.00, 3.84)]	1.95 ± 0.86 [1.99 (1.45, 2.10)]	0.005 *	0.366
90-HT	2.73 ± 0.54 [2.78 (2.28, 3.15)]	1.13 ± 0.45 [1.22 (0.96, 1.37)]	<0.001 **	3.68 ± 0.83 [3.32 (3.17, 4.39)]	1.63 ± 0.69 [1.56 (0.98, 2.12)]	<0.001 **	0.380
Profilometric outcomes							
ST0	5.25 ± 0.73 [5.38 (4.81, 5.82)]	4.29 ± 1.33 [4.59 (3.37, 5.15)]	0.094	5.05 ± 1.23 [5.16 (4.47, 5.47)]	3.92 ± 1.14 [3.61 (3.20, 4.15)]	0.078	0.683
ST1	5.75 ± 1.22 [5.44 (5.13, 5.77)]	4.95 ± 1.25 [4.68 (4.19, 5.13)]	0.215	5.90 ± 1.33 [6.00 (5.17, 6.91)]	4.63 ± 1.09 [4.60 (3.91, 5.55)]	0.056	0.388
ST2	5.12 ± 1.11 [5.11 (4.55, 5.66)]	4.41 ± 0.61 [4.17 (4.01, 4.69)]	0.134	5.55 ± 1.91 [5.49 (4.00, 6.75)]	5.01 ± 1.55 [4.61 (3.95, 5.74)]	0.544	0.642
45-ST	5.01 ± 0.91 [4.99 (4.51, 5.76)]	3.14 ± 0.73 [3.28 (2.77, 3.57)]	<0.001 **	4.85 ± 0.99 [4.77 (4.47, 5.10)]	3.29 ± 0.96 [3.28 (2.88, 3.50)]	0.007 *	0.511
90-ST	5.98 ± 1.46 [5.39 (4.90, 7.06)]	3.85 ± 1.17 [3.62 (3.19, 4.51)]	0.006 *	6.04 ± 1.84 [6.48 (4.56, 6.82)]	3.49 ± 0.71 [3.45 (3.05, 4.14)]	0.003 *	0.558

DH, defect height; DW, defect width; HT, hard-tissue profile at the implant shoulder (HT0), 1 mm below (HT1), 2 mm below (HT2), 45° (45-HT), and 90° (90-HT) positive angles; ST, soft tissue profile at the implant shoulder (ST0), 1 mm below (ST1), 2 mm below (ST2), 45° (45-ST), and 90° (90-ST) positive angles, respectively. Data are expressed as mean ± standard deviation [median, (first and third quartiles)]. *p*-value in the two comparison groups (* *p* ≤ 0.05 and ** *p* ≤ 0.001).

## Data Availability

The data supporting the findings of this study are not publicly available due to ethical and privacy restrictions, but are available from the corresponding author upon reasonable request.

## Abbreviations

The following abbreviations are used in this manuscript:

BBS	Block bone substitutes
SAT	Self-assembly technology
RCM	Resorbable collagen membrane
GBR	Guided bone regeneration
PCM	Physically crosslinked membrane
NCM	Non-crosslinked membrane
